# Silybin Mitigates Post-Myocardial Infarction Heart Failure in Mice via Modulation of HIF-1α-Driven Glycolysis and Energy Metabolism

**DOI:** 10.3390/nu17172800

**Published:** 2025-08-28

**Authors:** Mengyuan Wang, Jinhong Chen, Zhongzheng Zhang, Tianyu Wang, Jiaqi Zhao, Xiao Wang, Junyan Wang, Haowen Zhuang

**Affiliations:** 1 School of Basic Medical Sciences, Guangzhou University of Chinese Medicine, Guangzhou 510006, China; wmy8762@163.com (M.W.); 18002630878@163.com (J.C.); xwang72@gzucm.edu.cn (X.W.); 2 School of Pharmaceutical Sciences, Guangzhou University of Chinese Medicine, Guangzhou 510006, China; 18381497949@163.com (Z.Z.); 13091428177@163.com (T.W.); 13140096664@163.com (J.Z.)

**Keywords:** silybin, HIF-1α, glycolysis, energy metabolism disorder, heart failure

## Abstract

Background: Post-myocardial infarction (MI) heart failure (HF) is characterized by myocardial energy metabolism disorder, with excessive glycolysis playing a key role in its progression. Silybin (SIL), a flavonoid derived from Silybum marianum, has demonstrated hepatoprotective and metabolic regulatory effects. However, the role of this flavonoid in ameliorating post-myocardial infarction heart failure (post-MI HF) by modulating energy metabolism remains unclear. Methods: This study employed an oxygen–glucose deprivation (OGD) model to induce myocardial cell injury in vitro, with YC-1 treatment used to inhibit hypoxia-inducible factor-1α (HIF-1α) for mechanistic validation. A myocardial infarction-induced HF mouse model was used for in vivo experiments. Results: In vitro, SIL enhanced cell viability, increased ATP levels, and decreased lactate production and reactive oxygen species (ROS) accumulation in OGD-treated myocardial cells. SIL downregulated the mRNA and protein expression of HIF-1α, 6-phosphofructo-2-kinase/fructose-2,6-biphosphatase 3 (PFKFB3), glucose transporter 1 (GLUT1), and lactate dehydrogenase A (LDHA) while inhibiting HIF-1α nuclear translocation. Furthermore, SIL suppressed glycolytic proteins (PFKFB3, GLUT1, and LDHA) in a manner comparable to the HIF-1α inhibitor YC-1. This confirms that SIL’s inhibition of glycolysis is HIF-1α-dependent. In vivo, SIL treatment improved cardiac function parameters (LVEF and LVFS) and attenuated left ventricular remodeling (LVID;d and LVID;s) in post-MI HF mice. Additionally, myocardial fibrosis markers were significantly reduced, accompanied by a decrease in the myocardial mRNA and protein expression of glycolytic proteins, including HIF-1α, PFKFB3, GLUT1, and LDHA. Conclusions: Silybin effectively ameliorates post-myocardial infarction heart failure through the HIF-1α-mediated regulation of glycolysis, leading to improved myocardial energy metabolism and enhanced cardiac function.

## 1. Introduction

Heart failure (HF) is a complex clinical syndrome predominantly triggered by myocardial infarction (MI) secondary to ischemic heart disease. This condition constitutes a critical global health burden, affecting over 64 million individuals worldwide, with its prevalence continuing to rise due to population aging. The disease burden is particularly pronounced among elderly populations [1]. Myocardial infarction leads to the development and progression of heart failure through a cascade of ischemia/reperfusion injury, inflammatory responses, and myocardial fibrosis [2]. Despite therapeutic advances, including percutaneous coronary intervention and evidence-based pharmacotherapy, HF-associated mortality remains unacceptably high, constituting a major public health burden [3]. Consequently, elucidating the mechanisms underlying HF progression and identifying novel therapeutic targets are clinically imperative.

Accumulating evidence demonstrates that myocardial energy metabolism dysfunction is a critical determinant of HF progression [4]. Glycolysis, the metabolic pathway that converts glucose to pyruvate, is fundamental to cardiac energetics. Under physiological conditions, the heart derives approximately 95% of its energy from mitochondrial oxidative phosphorylation, with glycolysis contributing merely 5%. However, in HF, glycolytic flux increases as a compensatory response to sustain the provision of energy in the failing myocardium [5]. Hypoxia-inducible factor-1α (HIF-1α) functions as a master transcriptional regulator of cardiac glycolytic metabolism [6]. HIF-1α-mediated glycolytic reprogramming serves as a critical molecular foundation for the development and progression of pathological cardiac hypertrophy [7]. In addition, HIF-1α acts as a key transcriptional regulator of cellular glycolysis across diverse pathological conditions, ranging from respiratory diseases and infections to cancer [8,9]. Therefore, targeting the HIF-1α-mediated glycolytic pathway offers a potential therapeutic approach for restoring myocardial energy homeostasis, enhancing cardiac function, and reducing HF-associated mortality.

Silymarin (SIL), a bioactive flavonoid complex derived from *Silybum marianum*, is a clinically established hepatoprotective therapeutic agent. Previous studies have demonstrated that SIL enhances hepatic energy metabolism via multiple pathways, including suppression of NADPH oxidase expression and NF-κB signaling [10], activation of the AMPK/TGF-β1/Smad axis to ameliorate insulin resistance [11], and attenuation of hepatocellular injury. Notably, SIL exhibits potent inhibitory effects on hepatic glycolytic metabolism [12]. Nevertheless, the potential of SIL to restore energy metabolic homeostasis in post-MI HF through HIF-1α-mediated glycolytic modulation remains unexplored. Therefore, the primary aim of this study was to investigate the therapeutic mechanisms by which SIL ameliorates heart failure following myocardial infarction. For this purpose, we examined whether SIL exerts its cardioprotective effects through the modulation of HIF-1α-mediated glycolysis, with the goal of identifying novel therapeutic targets for post-MI heart failure management.

## 2. Materials and Methods

### 2.1. Drugs and Reagents

Silybin (purity ≥ 98%) was purchased from Sigma-Aldrich (St. Louis, MO, USA, S0417). High-glucose Dulbecco’s Modified Eagle Medium (DMEM, Gibco, Grand Island, NY, USA, 11965092,), penicillin/streptomycin (Thermo Fisher Scientific, Waltham, MA, USA, 15140122), ethylenediaminetetraacetic acid (EDTA, Gibco, Grand Island, NY, USA 25200056), fetal bovine serum (FBS, Gibco, Grand Island, NY, USA, A5670801), Evo M-MLV RT Premix for qPCR (Accurate Biology, Changsha, China, AG11706), a SYBR Green Premix Pro TaqHS qPCR Kit (Accurate Biology, Changsha, China, AG11718), RNAzol^®^ RT RNA extraction reagent (Mrcgene, OH, USA, RN190), Cell Counting Kit-8 (CCK-8, Yeasen Biotech, Shanghai, China, CX001S), an ATP Assay Kit (Beyotime, Shanghai, China, S0026B), a Lactate dehydrogenase assay kit (Nanjing Jiancheng Bioengineering Institute, Nanjing, China, A020-1-2), a Reactive Oxygen Species (ROS) Assay Kit (Beyotime, Shanghai, China, S0033S), YC-1 (Sigma-Aldrich, Y102), and a Nuclear and Cytoplasmic Protein Extraction Kit (Beyotime, Shanghai, China, P0027) were obtained from the indicated suppliers.

### 2.2. Animals and Ethics Statement

All animal experimental procedures were conducted in accordance with the Guidelines for the Care and Use of Laboratory Animals published by the National Institutes of Health (NIH Publication No. 85-23, revised 1996) and were approved by the Animal Ethics Committee of Guangzhou University of Chinese Medicine (approval number: ZYD-2024-041). This study was conducted and reported according to the ARRIVE guidelines for reporting animal research [13]. Fifty healthy male C57BL/6J mice (body weight: 20 ± 2 g) of specific pathogen-free (SPF) grade were procured from the supplier. Animals were housed under standardized conditions with a 12-h light/dark cycle, relative humidity of 55 ± 5%, and a constant temperature of 25 °C. All mice underwent a 3-day acclimatization period prior to experimental procedures. Ten mice were randomly allocated initially to each experimental group. After excluding postoperative deaths and animals with unsuccessful modeling, the final sample sizes for analysis were as follows: *n* = 6 (Sham), *n* = 6 (MI), *n* = 6 (SIL 100 mg/kg), *n* = 6 (SIL 200 mg/kg), and *n* = 6 (positive control). At study termination, animals were euthanized via intraperitoneal injection of pentobarbital sodium (150 mg/kg) followed by cervical dislocation.

### 2.3. Drug Preparation

Silybin powder (29.127 mg) was dissolved in 1 mL of sterile dimethyl sulfoxide (DMSO) to prepare a 50 mmol·L^−1^ stock solution. Following complete dissolution, the solution was sterilized by filtering it through a 0.22 μm membrane and kept at −20 °C until needed. For cellular experiments, the solution was serially diluted with a culture medium to achieve final concentrations of 50, 75, 100, and 150 μmol/L.

### 2.4. Cell Culture, Oxygen–Glucose Deprivation Model, and HIF-1α Inhibition

HL-1 cardiomyocytes were grown in high-glucose DMEM with 10% FBS and 1% penicillin/streptomycin and maintained at 37 °C in a humidified environment with 5% CO_2_. To establish the oxygen–glucose deprivation (OGD) model, cells were cultured until reaching approximately 80% confluence. The culture medium was then changed to one without glucose, and the cells were placed in a hypoxic incubator containing 1% oxygen, 5% carbon dioxide, and 94% nitrogen for a duration of 6 h.

For HIF-1α inhibition experiments, YC-1 (10 μmol/L) was used. Prior to OGD treatment, cells were pretreated with YC-1 dissolved in DMSO for 2 h under normoxic conditions [14]. The medium was then switched to a glucose-free one with YC-1, and the cells were exposed to hypoxic conditions as previously mentioned. DMSO (0.1%, *v*/*v*) served as the vehicle control.

### 2.5. Cell Viability Assay

HL-1 cells in the exponential growth phase were distributed into 96-well plates with a density of 5000 cells per well. Following 6 h of OGD treatment, cells were incubated with a complete culture medium containing silybin at concentrations of 50, 75, 100, and 150 μmol/L for 18 h. The CCK-8 assay was used to evaluate cell viability following the manufacturer’s guidelines, with absorbance readings taken at 450 nm using a multifunctional microplate reader from Thermo Scientific, Waltham, MA, USA.

### 2.6. Myocardial Infarction Model and Grouping

Fifty 8-week-old male wild-type mice were randomly allocated into five groups (*n* = 10 per group initially): a sham-operated group (sham), a myocardial infarction group (MI), silybin (SIL) 100 mg/kg group, an SIL 200 mg/kg group, and a positive control group. Male wild-type mice, aged eight weeks, were placed under anesthesia with 2% isoflurane and intubated using a ventilator designed for small animals. After performing a left thoracotomy through the fourth intercostal space, myocardial infarction was caused by permanently tying off the left anterior descending coronary artery using 8-0 sutures. Sham-operated animals underwent identical surgical procedures except for coronary artery ligation. The thoracic incision was subsequently closed in layers, and animals were placed on a heating pad during recovery. Due to the inherent mortality and modeling failure associated with left anterior descending (LAD) coronary artery ligation, the final sample size was *n* = 6 mice per group. Silybin treatment was initiated 5 weeks post-surgery, administered once daily via oral gavage for 4 consecutive weeks at doses of 100 or 200 mg/kg body weight, based on previous studies [15,16,17]. The sham-operated groups received equivalent volumes of saline following the same administration protocol.

As a positive control, fosinopril was suspended in a 0.5% carboxymethylcellulose sodium (CMC-Na) aqueous solution following, the same vehicle preparation protocol as the test groups, administered at 10 mg/kg body weight once daily via oral gavage for 4 consecutive weeks, starting concurrently with the test compound treatment. The fosinopril dosage was selected based on previous studies demonstrating significant cardioprotective effects in murine myocardial infarction models, where this dose was shown to effectively reduce cardiac fibrosis and improve cardiac function parameters including ejection fraction and fractional shortening [18].

### 2.7. Echocardiographic Assessment

Transthoracic echocardiography was performed to evaluate cardiac function. Mice were anesthetized with isoflurane gas following the removal of chest hair using depilatory cream. Acoustic coupling gel was applied uniformly, and optimal imaging angles were obtained using a high-frequency ultrasound probe designed for small animals. Echocardiographic parameters including left ventricular ejection fraction (LVEF), left ventricular fractional shortening (LVFS), left ventricular internal diameter at end-diastole (LVID;d), and left ventricular internal diameter at end-systole (LVID;s) were measured and analyzed. The relative wall thickness (RWT) was calculated using the following formula: RWT = (LVS;d + LVPW;d)/LVID;d. Four weeks post-surgery, an echocardiographic assessment was performed to evaluate cardiac function. Mice with an LVEF < 40% were classified as heart failure models and randomly allocated to four experimental groups, excluding a sham-operated group. Mice that died during surgery and those with an LVEF > 40% were excluded from further experimental analysis. Following four weeks of treatment, the echocardiographic assessment was repeated to evaluate therapeutic effects and document changes in cardiac parameters.

### 2.8. Histological Assessment

The cardiac tissues from mice were fixed using a 4% paraformaldehyde solution and later embedded in paraffin wax. Following standard protocols, 5 μm-thick serial tissue sections were prepared and stained with hematoxylin and eosin (H&E) and Masson’s trichrome. Mitochondrial morphology and ultrastructural analysis were performed using transmission electron microscopy (TEM, HT7800, Hitachi, Tokyo, Japan). The detailed data can be found in Figure S1.

### 2.9. Immunofluorescence Staining

After establishing the OGD model and administering silybin treatment, cells were fixed with 4% paraformaldehyde for 15 min at room temperature and rinsed three times with phosphate-buffered saline (PBS) for 10 min each. Cells underwent permeabilization with 0.2% Triton X-100 for 5 min, after which they were washed three times with PBS. A 5% BSA solution in PBS was applied for 30 min at room temperature to block non-specific binding. Frozen tissue sections were processed using the same protocol. Samples were incubated overnight at 4 °C with a primary antibody against HIF-1α (1:1000, Proteintech, Wuhan, China, 20960-1-AP). Following primary antibody incubation, samples were washed three times with PBS under light protection and incubated with fluorescent secondary antibodies (1:10,000, Proteintech, Wuhan, China, SA00001-1) for 45 min at room temperature. Three additional 10-min PBS washes were performed to remove unbound antibodies.

For ROS detection, unfixed cells from different experimental groups were directly incubated with an ROS-specific fluorescent probe (1:1000 dilution) for 30 min, according to the manufacturer’s instructions. Nuclear counterstaining was performed using 4′,6-diamidino-2-phenylindole (DAPI) for 3 min. Samples were mounted with 95% glycerol and examined under fluorescence microscopy.

### 2.10. ATP and Lactic Acid Quantification

Cell culture supernatants were collected along with mouse serum samples for subsequent biochemical analysis. ATP and lactic acid concentrations were determined using commercially available assay kits according to the manufacturers’ protocols. Samples were processed in designated microplate wells, and measurements were performed using appropriate spectrophotometric methods. Metabolite concentrations were calculated from standard curves and normalized to the total protein concentration or cell number as appropriate.

### 2.11. Real-Time Quantitative Polymerase Chain Reaction (RT-qPCR)

Total RNA was extracted from cardiomyocytes and mouse cardiac tissues using an RNAzol reagent according to standard protocols. RNA integrity and concentration were determined spectrophotometrically. Complementary DNA (cDNA) synthesis was performed using Evo M-MLV RT Premix following standard procedures. Quantitative real-time PCR was executed on a QuantStudio 5 Real-Time PCR System utilizing SYBR Green chemistry. The 2^ΔΔCt^ method was used to calculate relative gene expression, with β-actin as the control gene.

### 2.12. Western Blotting Analysis

Using a RIPA lysis buffer with protease inhibitors, total protein was extracted from cardiac tissues and cultured heart cells, and protein levels were determined using a BCA Protein Assay Kit. Protein samples ranging from 30 to 50 μg were separated using 10% SDS-PAGE and then transferred onto PVDF membranes (Thermo Fisher Scientific, Waltham, MA, USA, Cat# 88518). The membranes were blocked with 5% non-fat milk in TBST for one hour at room temperature. Primary antibodies were incubated overnight at 4 °C at the indicated dilutions: HIF-1α (1:1000, Proteintech, Wuhan, China, 20960-1-AP), PFKFB3 (1:1000, Abcam, Cambridge, Cambridgeshire, UK, ab181861), GLUT1 (1:1000, Proteintech, Wuhan, China, 21829-1-AP), LDHA (1:1000, Proteintech, Wuhan, China, 19987-1-AP), AMPK (1:1000, Proteintech, Wuhan, China, 10929-2-AP), and α-sma (1:2000, Proteintech, Wuhan, China, 14395-1-AP). After washing, membranes were incubated with secondary antibodies. Following the washing step, membranes were treated with secondary antibodies. Protein bands were identified through enhanced chemiluminescence, and their density was quantified using the ImageJ 1.8.0 software.

### 2.13. Network Pharmacology Analysis

#### 2.13.1. Construction of Drug-Disease Common Targets

Active chemical components of silybin were identified from the Traditional Chinese Medicine Systems Pharmacology (TCMSP) database using the selection criteria of oral bioavailability (OB) ≥ 30% and drug-likeness (DL) ≥ 0.18. The selected compounds were imported into the UniProt database (https://www.uniprot.org/, accessed on 20 September 2024) to obtain corresponding target proteins, which were subsequently mapped to gene names with species restricted to “Homo sapiens”. These targets were then imported into the Cytoscape 3.9.1 software to construct comprehensive drug-component-target networks. Heart failure-related genes were identified through systematic searches of the GeneCards (https://www.genecards.org/, accessed on 20 September 2024) and Online Mendelian Inheritance in Man (OMIM, https://omim.org/, accessed on 20 September 2024) databases using “heart failure” as the search term. Venn diagram analysis was performed to identify overlapping genes between silybin targets and heart failure-associated genes.

#### 2.13.2. Construction of PPI Network and Identification of Hub Targets

The protein–protein interaction (PPI) network analysis was conducted using the STRING 11.5 platform (https://string-db.org/, accessed on 24 September 2024). The intersecting genes were uploaded with the species set to “Homo sapiens” and the minimum interaction threshold adjusted to “medium confidence” (0.4). Disconnected nodes were hidden while all other parameters remained at default settings. The Cytoscape software (v3.8.2) was utilized to calculate the topological parameters including degree centrality, betweenness centrality, and closeness centrality to identify hub genes and core targets.

#### 2.13.3. GO and KEGG Pathway Enrichment Analysis

Gene Ontology (GO) functional annotation and Kyoto Encyclopedia of Genes and Genomes (KEGG) pathway enrichment analyses were performed using the Database for Annotation, Visualization and Integrated Discovery (DAVID, https://david.ncifcrf.gov/summary.jsp, accessed on 29 September 2024) to elucidate potential molecular mechanisms and signaling pathways involved in silybin-mediated cardioprotection.

### 2.14. Molecular Docking Analysis

Three-dimensional protein structures were obtained from the Protein Data Bank (PDB), and ligand structures were optimized prior to docking calculations. The molecular interaction between HIF-1α and silybin was then evaluated through computational molecular docking using the AutoDock Vina 1.2.5 software. The binding affinity values (kcal/mol) represent the calculated binding free energy, where more negative values indicate greater thermodynamic stability of the ligand–receptor complex.

### 2.15. Statistical Analysis

The GraphPad Prism 9 software (GraphPad Software, San Diego, CA, USA) was used to conduct statistical analyses. The results are expressed as the mean ± standard deviation (SD) from at least three independent trials. Data normality was first assessed using the Shapiro–Wilk test. For comparing two groups, Student’s *t*-test was used. Multiple group comparisons were performed using a one-way ANOVA with Tukey’s post hoc test. For non-normally distributed data, the Kruskal–Wallis test was used. The statistical significance level was set at *p* < 0.05.

## 3. Results

### 3.1. SIL Ameliorates Myocardial Cell Injury

**Figure 1:**
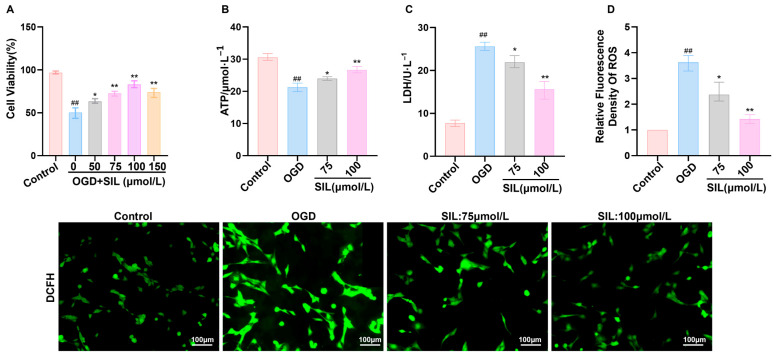
SIL ameliorates myocardial cell injury. (**A**) CCK-8 assay in HL-1 cells treated with SIL (0–150 μM). (**B**) ATP level determination. (**C**) Lactate level determination. (**D**) Immunofluorescence detection of ROS levels. Scale bar = 100 μm. ^##^ *p* < 0.01 vs. control; * *p* < 0.05, ** *p* < 0.01 vs. OGD; (*n* = 3).

To examine the cardioprotective mechanism of SIL in heart failure, an oxygen–glucose deprivation (OGD) model was established to simulate myocardial injury in vitro. Cell viability assays demonstrated that OGD treatment markedly reduced cardiomyocyte survival compared with the controls (*p* < 0.01). SIL treatment exhibited dose-dependent protection, with enhanced cell viability observed across all tested concentrations (50–150 μmol/L, *p* < 0.05) (Figure 1A). Based on optimal protective efficacy, 75 μmol/L and 100 μmol/L concentrations were selected for mechanistic investigations. Bioenergetic analysis showed that OGD treatment decreased ATP production (*p* < 0.01) and increased lactate dehydrogenase (LDH) release (*p* < 0.01), reflecting metabolic impairment and membrane damage. SIL treatment (75 and 100 μmol/L) restored ATP levels (*p* < 0.05) and reduced LDH leakage (*p* < 0.05) (Figure 1B,C). The immunofluorescence analysis revealed intense cytoplasmic fluorescence following OGD exposure, consistent with oxidative stress. SIL treatment (75 and 100 μmol/L) reduced fluorescence intensity, confirming the effective mitigation of oxidative damage (Figure 1D).

### 3.2. SIL Engages with the HIF-1α Signaling Pathway Based on Network Pharmacology and MOD

**Figure 2:**
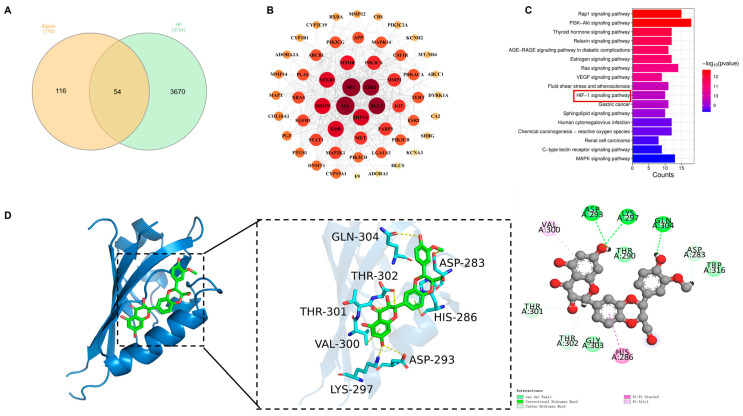
SIL targeting the HIF-1α signaling pathway through a network pharmacology and molecular docking analysis. (**A**) Venn diagram. (**B**) PPI network. (**C**) KEGG. HIF-1α signaling pathway was identified as the core pathway. (**D**) Molecular docking between SIL and HIF-1α.

To elucidate the molecular mechanisms underlying the protective effects of SIL against HF dysfunction, network pharmacology was integrated with molecular docking analysis. This approach identified 54 overlapping targets from 170 SIL-associated proteins and 3724 HF-related genes (Figure 2A). PPI networks and topological characteristics were constructed and analyzed using Cytoscape 3.9.1 (Figure 2B). Topological analysis of the PPI network identified HIF-1α as the predominant hub node, which exerts its central regulatory function in the protein interaction network (Degree = 32). KEGG pathway enrichment analysis revealed that SIL attenuates HF dysfunction through multiple cellular pathways, particularly the HIF-1α signaling pathway (Figure 2C). Molecular docking demonstrated strong binding affinity between SIL and HIF-1α (binding energy: −5.9916 kcal/mol), with key interactions at residues GLN-304, THR-302, THR-301, ASP-283, HIS-286, VAL-300, ASP-293, and LYS-297 (Figure 2D). These results suggest that SIL may preserve cellular homeostasis through HIF-1α-mediated glycolytic pathways.

### 3.3. SIL Regulates HIF-1α-Mediated Glycolytic Gene Expression In Vitro

**Figure 3:**
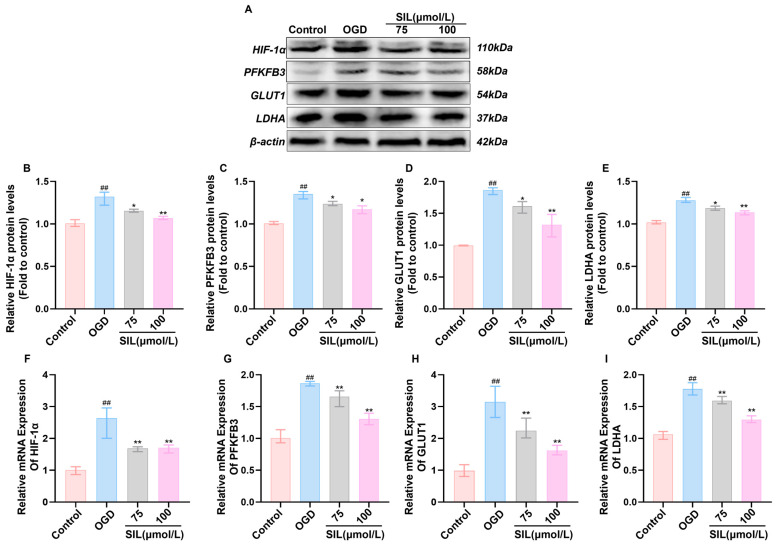

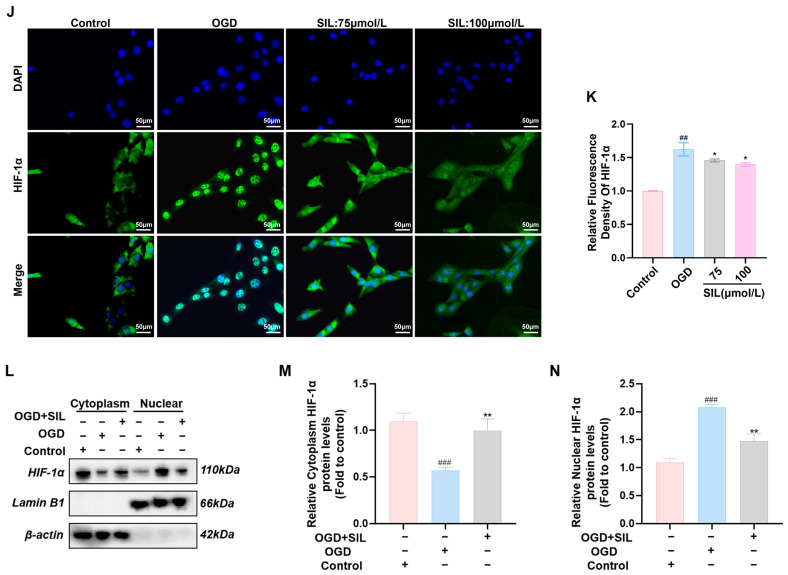
SIL regulates HIF-1α-mediated glycolytic gene expression in vitro. (**A**) Western blot analysis of HIF-1α, PFKFB3, GLUT1, and LDHA protein expression (*n* = 3). (**B**–**E**) Quantitative analysis of relative protein expression levels of HIF-1α, PFKFB3, GLUT1, and LDHA. (**F**–**I**) qRT-PCR analysis of HIF-1α, GLUT1, PFKFB3, and LDHA mRNA expression (*n* = 6). (**J**,**K**) Fluorescence expression intensity and relative expression levels of HIF-1α in HL-1. Scale bar = 50 μm (*n* = 3). (**L**) Western blot analysis of HIF-1α protein expression in cytoplasmic and nuclear fractions following nuclear-cytoplasmic separation (*n* = 3). (**M**,**N**) Quantitative analysis of relative cytoplasmic and nuclear HIF-1α protein expression levels (*n* = 3). ^##^ *p* < 0.01, ^###^ *p* < 0.001 vs. control; * *p* < 0.05, ** *p* < 0.01 vs. OGD.

Based on the network pharmacology results, SIL potentially exerts protective effects by regulating the HIF-1α signaling pathway. An oxygen–glucose deprivation (OGD)-induced myocardial cell injury model was employed to investigate the impact of SIL on HIF-1α-mediated glycolysis. Compared with the control group, OGD treatment significantly increased mRNA and protein expression of key glycolytic enzymes including HIF-1α, PFKFB3, GLUT1, and LDHA in HL-1 (*p* < 0.01) (The specific primer sequences are detailed in Table 1). SIL intervention dose-dependently attenuated the expression of these molecules (*p* < 0.05) (Figure 3A–I), and an immunofluorescence analysis confirmed these findings. HIF-1α fluorescence intensity was markedly elevated in the OGD group, while SIL treatment produced a concentration-dependent reduction in fluorescence signals (Figure 3J). These results demonstrate that SIL modulates HIF-1α pathway-associated glycolytic enzyme expression and contributes to myocardial cell protection against ischemic injury.

Nuclear/cytoplasmic protein fractionation was used to evaluate HIF-1α distribution between cytoplasm and the nucleus. OGD treatment significantly increased nuclear HIF-1α levels (*p* < 0.001) compared with the controls, while cytoplasmic levels decreased (*p <* 0.001), indicating the hypoxia-induced nuclear translocation of HIF-1α. Silybin treatment (100 μmol/L) significantly reduced nuclear HIF-1α levels compared with those in the OGD group (*p* < 0.01) and increased cytoplasmic levels (*p* < 0.01) (Figure 3L–N). These results demonstrate that silybin inhibits HIF-1α nuclear translocation.

### 3.4. SIL Repairs OGD-Induced Myocardial Cell Injury Through HIF-1α-Mediated Glycolytic Pathway

**Figure 4:**
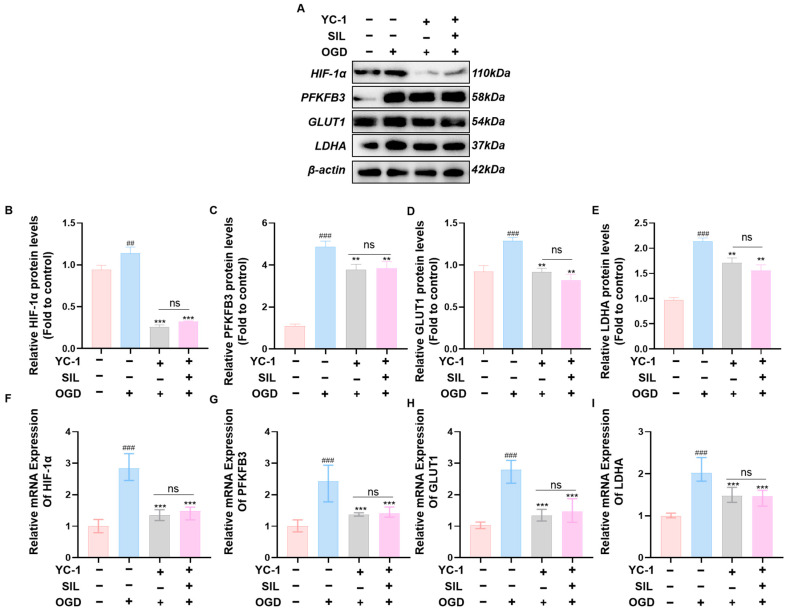
SIL regulates the glycolytic signaling pathway via HIF-1α. (**A**–**E**) Western blot analysis and quantification of HIF-1α, PFKFB3, GLUT1, and LDHA protein expression after the administration of an HIF-1α inhibitor (*n* = 3). (**F**–**I**) qRT-PCR analysis of HIF-1α, GLUT1, PFKFB3, and LDHA mRNA expression (*n* = 6). ^##^ *p* < 0.01, ^###^ *p* < 0.001 vs. control; ** *p* < 0.01, *** *p* < 0.001 vs. OGD. ns, OGD+YC-1 vs. OGD+YC-1+SIL.

Western blot analysis showed that OGD treatment significantly increased HIF-1α protein expression compared with the control group (*p* < 0.01). YC-1 treatment markedly reduced HIF-1α expression in the OGD+YC-1 group compared with the OGD group (*p* < 0.001). The OGD+YC-1+SIL group also showed significantly decreased HIF-1α protein expression compared with that in the OGD group (*p* < 0.001). HIF-1α protein levels showed no significant difference between the OGD+YC-1 and OGD+YC-1+SIL groups (Figure 4A,B). Protein levels of glycolytic enzymes showed similar patterns. PFKFB3, GLUT1, and LDHA were significantly decreased in the OGD+YC-1 group compared with those in the OGD group (*p* < 0.01). These glycolytic enzymes also remained significantly lower in the OGD+YC-1+SIL group compared with those in the OGD group (*p* < 0.01). No significant differences were observed between the OGD+YC-1 and OGD+YC-1+SIL groups (Figure 4A,C–E).

RT-qPCR analysis showed that OGD treatment significantly increased HIF-1α mRNA expression compared with that in the control group (*p* < 0.001) (Figure 4F). YC-1 treatment markedly reduced HIF-1α mRNA in the OGD+YC-1 group compared with that in the OGD group (*p* < 0.001). The OGD+YC-1+SIL group also showed significantly lower HIF-1α mRNA levels compared with those in the OGD group (*p* < 0.001). There was no significant difference in HIF-1α mRNA expression between the OGD+YC-1 and OGD+YC-1+SIL groups. Similarly, the mRNA levels of key glycolytic enzymes PFKFB3, GLUT1, and LDHA were significantly elevated in the OGD group compared with those in the control (*p* < 0.001) (Figure 4G–I). YC-1 treatment decreased these glycolytic gene expressions in the OGD+YC-1 group compared with those in the OGD group (*p* < 0.001). The OGD+YC-1+SIL group also maintained significantly lower levels of PFKFB3, GLUT1, and LDHA mRNA compared with those in the OGD group (*p* < 0.001). No significant differences were observed in these glycolytic gene expressions between the OGD+YC-1 and OGD+YC-1+SIL groups. These results indicate that YC-1-mediated inhibition of HIF-1α blocks the regulatory effects of SIL on glycolytic gene expression.

### 3.5. SIL Improves Cardiac Function in Mice with Heart Failure After Myocardial Infarction

To investigate the cardioprotective effects of SIL against MI-induced cardiac injury, in vivo experiments were performed to evaluate cardiac function, myocardial tissue morphology, and fibrosis markers. Echocardiographic analysis revealed that MI significantly impaired cardiac function compared with the sham group, characterized by substantial decreases in LVEF and LVFS (*p* < 0.001) and significant increases in LVID;d and LVID;s (*p* < 0.001), indicating cardiac systolic dysfunction and ventricular dilation.

**Figure 5:**
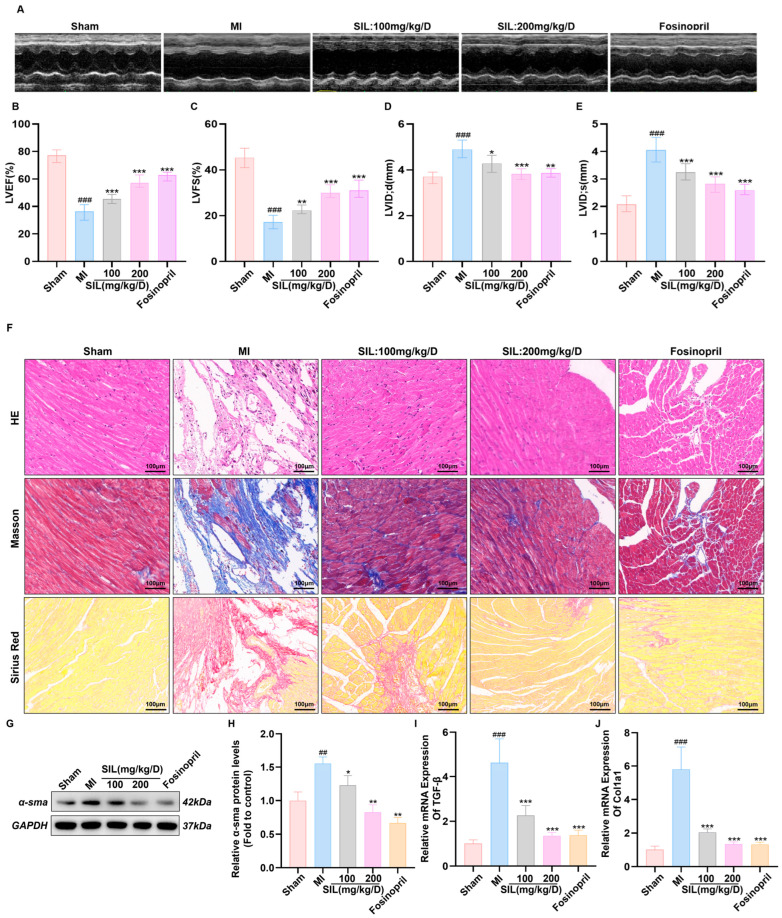
SIL improves cardiac function and myocardial remodeling in mice with heart failure after myocardial infarction. (**A**) Representative echocardiographic images. (**B**–**E**) Quantitative analysis of echocardiographic parameters including LVEF, LVFS, LVID;d, and LVID;s (*n* = 6). (**F**) Representative images of right atrial sections stained with hematoxylin-eosin (HE), Masson trichrome, and Sirius red, scale bar = 100 μm. (**G**,**H**) Western blot analysis and quantification of α-sma protein expression. (**I**,**J**) qRT-PCR analysis of TGF-β and Col1a1 mRNA expression (*n* = 6). ^##^ *p* < 0.01, ^###^ *p* < 0.001 vs. sham; * *p* < 0.05, ** *p* < 0.01, *** *p* < 0.001 vs. MI; (*n* = 3).

Both SIL treatment (100 and 200 mg/kg/d) and fosinopril (positive control) dose-dependently improved cardiac function parameters. LVEF and LVFS were significantly increased in the SIL 100 mg/kg/d, SIL 200 mg/kg/d, and fosinopril groups compared with the values in the MI group (*p* < 0.01) (Figure 5B,C). Similarly, LVID;d and LVID;s were markedly decreased in all treatment groups (*p* < 0.05) (Figure 5D,E). These findings confirm the dose-dependent protective effects of SIL against MI-induced cardiac dysfunction (Figure 5A–E).

Histological examination via H&E staining revealed regular myocardial fiber arrangement in the sham group, whereas the MI group showed fiber disruption, dissolution, extensive inflammatory infiltration, and necrotic foci. Both SIL treatment (100 and 200 mg/kg/d) and fosinopril reduced myocardial fiber injury, decreased necrotic areas, and restored tissue morphology toward normal, with fosinopril yielding the best preservation of myocardial structures (Figure 5F). Masson’s trichrome staining showed extensive myocardial interstitial collagen deposition (blue staining) in the MI group, indicating myocardial fibrosis. Treatment with SIL and fosinopril significantly reduced collagen deposition, with fosinopril demonstrating the most effective antifibrotic effects, followed by SIL 200 mg/kg/d and SIL 100 mg/kg/d in a dose-dependent manner (Figure 5F). The results from Sirius Red staining indicated that myocardial tissue in the model group showed a significant increase in collagen fiber deposition compared with that in the sham group. Additionally, compared with the model group, there was a significant reduction in collagen fiber deposition within the myocardial tissue of the SIL treatment groups (100 mg/kg and 200 mg/kg), with a more pronounced effect observed in the 200 mg/kg group (Figure 5F).

Western blotting analysis showed that α-SMA expression, a key marker of myofibroblast activation and cardiac fibrosis, was significantly increased in the MI group (*p* < 0.01). SIL treatment effectively reduced α-SMA expression in a dose-dependent manner, with both treatment groups showing significant inhibition compared with the results in the MI group (*p* < 0.05) (Figure 5G,H), further confirming the antifibrotic properties of SIL. Consistently, qPCR analysis showed the mRNA levels of TGF-β1 and Col1a1 to be significantly elevated in the MI group (*p* < 0.001). Following SIL intervention, both TGF-β1 and Col1a1 mRNA levels were significantly downregulated (*p* < 0.001), suggesting that silybin plays a beneficial role in ameliorating fibrosis in MI mice (Figure 5I,J).

### 3.6. SIL Modulates HIF-1α-Mediated Glycolysis in Myocardial Tissue

**Figure 6:**
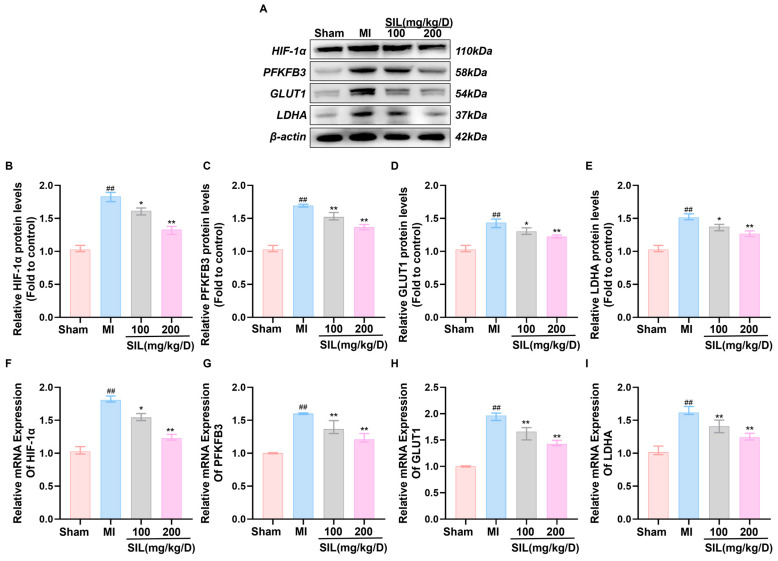

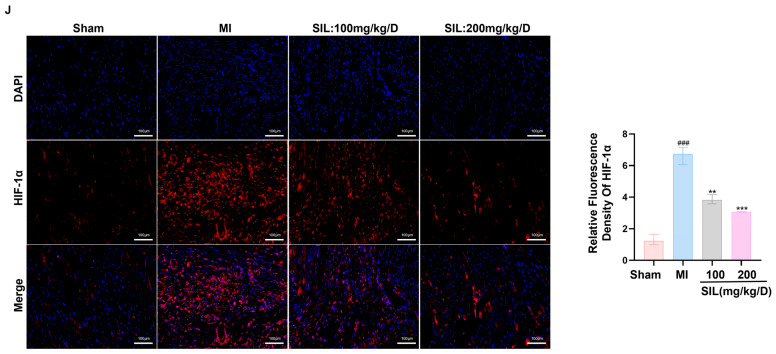
SIL modulates the HIF-1α-mediated glycolytic signaling pathway in vivo. (**A**) Western blot analysis of HIF-1α, PFKFB3, GLUT1, and LDHA protein expression (*n* = 3). (**B**–**E**) Quantitative analysis of the relative protein expression levels of HIF-1α, PFKFB3, GLUT1, and LDHA (*n* = 3). (**F**–**I**) qRT-PCR analysis of HIF-1α, GLUT1, PFKFB3, and LDHA mRNA expression (*n* = 6). (**J**) Fluorescence intensity and relative expression of HIF-1α in cardiomyocytes (*n* = 3), scale bar = 100 μm. ^##^ *p* < 0.01, ^###^ *p* < 0.01 vs. sham; * *p* < 0.05, ** *p* < 0.01, *** *p* < 0.01 vs. MI.

To investigate the effects of SIL on HIF-1α-regulated glycolytic signaling in vivo, myocardial tissue was analyzed for glycolytic enzyme expression. Western blotting analysis revealed significantly upregulated protein expression of HIF-1α, PFKFB3, GLUT1, and LDHA in the MI group compared with the values in the sham group (*p* < 0.01), indicating glycolytic pathway activation following MI. SIL treatment (100 and 200 mg/kg/d) dose-dependently reduced these protein levels, with the 100 mg/kg/d group showing partial reduction (*p* < 0.05), and the 200 mg/kg/d group demonstrating greater efficacy (*p* < 0.01) (Figure 6A–E). RT-qPCR analysis confirmed significantly elevated mRNA expression of HIF-1α, PFKFB3, GLUT1, and LDHA in the MI group compared with that in the sham group (*p* < 0.01). SIL treatment dose-dependently downregulated the mRNA expression of these glycolytic genes (*p* < 0.05), with optimal effects observed in the 200 mg/kg/d group (Figure 6F–I), suggesting transcriptional-level regulation.

Immunofluorescence analysis revealed weak HIF-1α fluorescence in the sham myocardial tissue, whereas the MI group exhibited significantly enhanced HIF-1α fluorescence intensity (*p* < 0.001), indicating MI-induced HIF-1α activation and nuclear translocation. SIL treatment dose-dependently reduced HIF-1α fluorescence intensity. The 100 mg/kg/d group showed a significant reduction than that in the MI group (*p* < 0.01), while the 200 mg/kg/d group demonstrated more pronounced effects (*p* < 0.001). Furthermore, SIL treatment restored HIF-1α distribution primarily to the cytoplasm, while nuclear fluorescence was markedly reduced in the SIL-treated group. These results indicate that SIL inhibits MI-induced HIF-1α activation and prevents its nuclear translocation (Figure 6J).

### 3.7. SIL Enhances Mitochondrial Structure and Function in Myocardial Tissue

**Figure 7:**
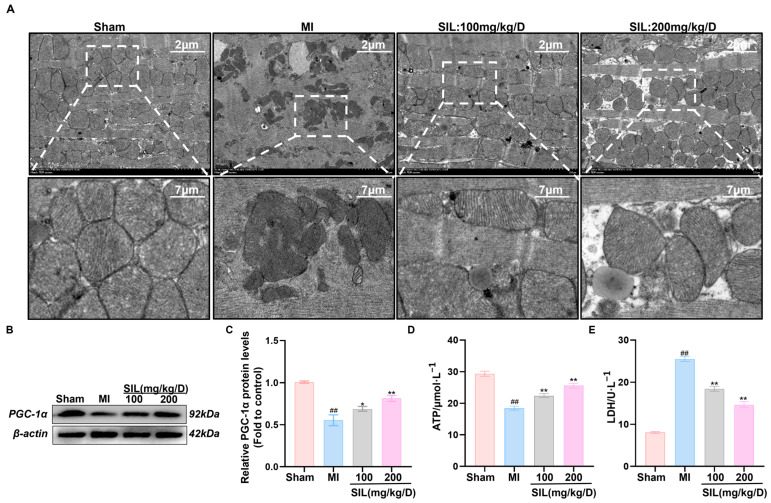
Silybin improves myocardial mitochondrial structure and function. (**A**) Transmission electron micrographs of mitochondria. (**B**,**C**) Western blot analysis and quantification of PGC-1α protein expression. (**D**) ATP content. (**E**) Lactic acid content. ^##^ *p* < 0.01, vs. sham; * *p* < 0.05, ** *p* < 0.01 vs. MI; (*n* = 3).

To elucidate the silybin (SIL)-mediated effects on myocardial energy metabolism after myocardial infarction (MI), we conducted experiments to assess the mitochondrial ultrastructure, pivotal energy metabolism molecules, and metabolic products. Transmission electron microscopy showed that compared with those in the Sham group, myocardial mitochondria in the MI group were less numerous, with their cristae fractured or absent. After SIL treatment, compared with the results for the MI group, the mitochondrial quantity increased, structural integrity improved significantly, and the high-dose group outperformed the low-dose group in restoration efficacy (Figure 7A).

Western blotting analysis showed that PGC-1α protein levels in the MI group were significantly lower than those in the Sham group (*p* < 0.01). After SIL intervention, PGC-1α protein expression in the myocardial tissue of low-dose and high-dose groups increased significantly (*p* < 0.05), confirming the SIL-mediated regulation of relevant protein expression (Figure 7B,C).

As shown by the ATP and LDH assays (Figure 7D,E), MI group ATP content was significantly lower than that in the Sham group (*p* < 0.01), while LDH levels were higher (*p* < 0.01). After SIL intervention, the low- and high-dose groups showed significantly increased ATP and decreased LDH when compared with the MI group (*p* < 0.01). The high-dose group exhibited greater efficacy in elevating ATP and reducing LDH than the low-dose group, further suggesting that SIL improves mitochondrial function via the glycolytic signaling pathway, exerting a favorable impact on myocardial cell energy metabolism processes.

## 4. Discussion

Heart failure (HF) represents a critical global health burden, affecting over 64 million individuals worldwide. The prevalence of this disease continues to rise with the aging population, exemplified by China where rates among adults ≥65 years increased from 1.3% (2000) to 3.86% (2017), reaching 7.55% in octogenarians [1,19,20]. This complex syndrome, characterized by dyspnea, edema, and fatigue, severely compromises quality of life while imposing substantial societal costs, highlighting the urgent need for improved therapeutic strategies.

Energy metabolism dysregulation drives HF pathogenesis. While a healthy heart relies predominantly on mitochondrial oxidative metabolism, pathological remodeling triggers reprogramming from efficient fatty acid oxidation toward inefficient glycolysis, depleting bioenergetic reserves and increasing glucose dependence [5,21]. This shift inadequately meets cardiac energy demands in end-stage HF, exacerbating mitochondrial dysfunction and contractile impairment while inducing ROS overproduction, oxidative stress, and inflammation [22,23]. Targeting this metabolic dysregulation, therefore, represents a promising therapeutic approach.

Silymarin (SIL), a flavonolignan complex from Silybum marianum seeds, was clinically established to treat hepatoprotection through antioxidative, anti-inflammatory, and antifibrotic mechanisms [24,25,26]. Emerging evidence, however, demonstrates its broader therapeutic potential. SIL modulates metabolic reprogramming by reducing triglyceride accumulation in the liver, ameliorates insulin resistance through the IRS-1/PI3K/Akt pathways, and provides cardioprotection by attenuating injury markers while inhibiting apoptotic signaling [27,28,29]. Despite improving glucose–lipid metabolism in metabolic syndrome [30], the mechanisms of SIL in HF remain incompletely understood. This study investigated the effects of SIL using complementary in vitro (OGD-treated HL-1 cardiomyocytes) and in vivo (LAD ligation-induced myocardial infarction) models.

The network pharmacology analysis revealed that IL6 (Degree = 38), SRC (Degree = 37), BCL2 (Degree = 33), and ESR1 (Degree = 33) exhibited slightly higher node connectivity than that of HIF-1α (Degree = 32). Despite the increased connectivity of IL6 and SRC, their related signaling pathways did not reach statistical significance in the KEGG enrichment analysis (*p* > 0.05) and showed limited hierarchical mechanistic relevance. Conversely, HIF-1α functions as a key transcriptional regulator of hypoxic metabolic reprogramming and directly influences the expression of inflammatory factors [31,32], processes that are intricately associated with the complex pathogenesis of heart disease. Importantly, hierarchical pathway analysis identified PI3K/AKT signaling as a canonical upstream regulator that modulates the stability and activity of HIF-1α through phosphorylation in disease models such as endometrial cancer, and asthma [33,34]. Moreover, the activation of Hif-1α upregulates the expression of vascular endothelial growth factor (VEGF), thereby mitigating apoptosis and facilitating the proliferation of endothelial cells [35]. Consequently, Hif-1α is crucial as the main therapeutic target for SIL in managing heart failure.

Importantly, these diverse functions converge on the regulation of energy metabolism homeostasis, which is a fundamental factor in the progression of heart failure. In particular, the dysregulation of energy metabolism, especially the pathological shift from oxidative phosphorylation to glycolysis, constitutes a central mechanism underlying the advancement of heart failure. HIF-1α serves as the master transcription factor orchestrating this metabolic reprogramming under hypoxic conditions [36]. Under pathological conditions, HIF-1α activation promotes the upregulation of key glycolytic enzymes (GLUT1, PFKFB3, LDHA) while suppressing mitochondrial biogenesis through PGC-1α inhibition [37,38,39]. Given the established metabolic regulatory properties of SIL, we hypothesized that SIL might confer cardioprotection by modulating the HIF-1α-glycolysis axis.

To test this hypothesis, the effects of SIL were evaluated in HL-1 cardiomyocytes subjected to OGD injury, an established myocardial ischemia model. SIL treatment preserved cell viability while markedly suppressing HIF-1α expression and downstream glycolytic targets (GLUT1, PFKFB3, LDHA), indicating cardioprotective effects involve HIF-1α-mediated glycolytic modulation.

These HIF-1α-regulated enzymes exhibit distinct roles in pathological cardiac metabolism. GLUT1 facilitates glucose uptake, with HIF-1α-mediated upregulation enhancing substrate availability during hypoxia [40]. PFKFB3 generates fructose-2,6-bisphosphate, which potently activates PFK-1—the rate-limiting glycolytic enzyme—thereby accelerating metabolic flux [41,42]. LDHA converts pyruvate to lactate, maintaining NAD+ regeneration under hypoxic conditions while promoting cellular acidification when excessive [43,44]. This coordinated enzymatic network illustrates how HIF-1α orchestrates comprehensive metabolic reprogramming in cardiac pathology.

To confirm HIF-1α’s role in mediating SIL’s effects, we performed loss-of-function experiments. In OGD-treated HL-1 cells, the HIF-1α inhibitor YC-1 mirrored the effects of SIL on glycolysis. Both interventions lowered HIF-1α and decreased the mRNA and protein levels of the glycolytic targets PFKFB3, GLUT1, and LDHA. Co-treatment with SIL and YC-1 yielded no additional suppression compared with YC-1 alone. These findings indicate that SIL regulates glycolysis primarily via an HIF-1α-dependent pathway. From the standpoint of regulatory mechanisms, both the mRNA and protein levels of HIF-1α demonstrated a synergistic reduction following silybin (SIL) treatment. This observation suggests that the underlying regulatory mechanism is more likely to involve the transcriptional regulation or modulation of mRNA stability, rather than relying solely on post-translational degradation processes. Additional evidence indicates that SIL significantly impedes the nuclear translocation of HIF-1α—a necessary condition for its transcriptional activity—while simultaneously down-regulating its downstream glycolytic target genes (GLUT1, PFKFB3, LDHA) at the mRNA level. These findings suggest that inhibiting the transcriptional activity of HIF-1α constitutes a critical regulatory node through which SIL exerts its metabolic regulatory effects.

In vivo validation in LAD ligation-induced HF mice corroborated these findings. Failing hearts exhibited elevated HIF-1α expression with upregulated glycolytic enzymes (GLUT1, PFKFB3, LDHA) and mitochondrial dysfunction. SIL reduced HIF-1α, GLUT1, PFKFB3, and LDHA at both the mRNA and protein levels, thereby restraining glycolysis. Subsequently, SIL partially restored mitochondrial oxidative capacity. Functionally, SIL-treated mice demonstrated an improved ejection fraction, reduced ventricular dilation, and attenuated cardiac fibrosis. These results indicate that SIL improves myocardial energy metabolism in vivo by inhibiting HIF-1α–mediated glycolysis, thereby alleviating heart failure.

Previous investigations have demonstrated that inhibiting HIF-1α or its downstream targets reduces excessive glycolysis and improves cardiac function in myocardial ischemia-reperfusion injury [45]. However, no previous studies have investigated whether SIL exerts its cardioprotective effects by modulating the HIF-1α pathway. The present study shows that SIL exerts cardioprotective effects by modulating the HIF-1α-glycolytic pathway, leading to the downregulation of key glycolytic enzymes (GLUT1, PFKFB3, LDHA) and the restoration of metabolic homeostasis in heart failure.

## 5. Limitations

This study has several limitations. First, our mechanistic investigation focused exclusively on the HIF-1α–glycolysis axis. Although we identified this pathway as a key mediator, SIL exhibits a complex pharmacological profile. Other signaling networks—including oxidative stress, ferroptosis, and inflammatory pathways—may also contribute to this pathway’s cardioprotective effects. Our analysis of HIF-1α’s upstream regulation was incomplete, with key mediators such as AMPK and ROS signaling remaining unexplored. Future studies should adopt a systems-based biological approach to elucidate more comprehensive mechanistic insights. Second, our findings are limited to preclinical models. These models may not fully reflect the pathophysiological complexity of human heart failure. Clinical translation faces additional challenges due to the poor oral bioavailability of SIL, whose high lipophilicity and low aqueous solubility may prevent medical professionals from achieving therapeutic myocardial concentrations. Novel formulations show promise in preclinical studies but require clinical validation in heart failure patients.

## 6. Conclusions

**Figure 8:**
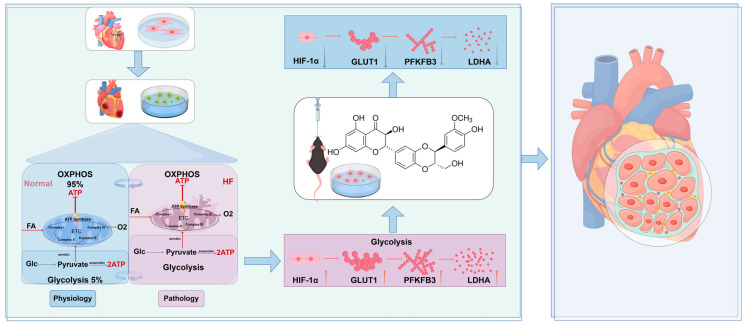
Potential mechanism of silybin’s protective role against post-myocardial infarction heart failure. Silybin downregulates the expression of HIF-1α, inhibits the nuclear translocation of HIF-1α, and reduces the expression of glycolytic-related proteins (PFKFB3, GLUT1, LDHA) mediated by HIF-1α, thereby improving myocardial energy metabolism disorder, reducing myocardial fibrosis and mitochondrial damage, and ultimately enhancing cardiac function. Created using https://www.figdraw.com (accessed on 14 April 2025) ( authorization code:ASIOS99490).

In this study, silybin was shown to alleviate post-myocardial infarction heart failure by downregulating HIF-1α expression and inhibiting its nuclear translocation, which, in turn, reduces the HIF-1α-mediated upregulation of key glycolytic enzymes (PFKFB3, GLUT1, LDHA). This process results in improved myocardial energy metabolism and mitigates mitochondrial damage and fibrosis. These findings underscore the potential of silybin as a therapeutic agent for targeting metabolic reprogramming in heart failure treatment (Figure 8).

## Figures and Tables

**Table 1:** The sequences of the mouse gene-specific primers required for RT-qPCR reaction.

Gene	Forward Primer (5′-3′)	Reverse Primer (5′-3′)
HIF-1α	GTCGGACAGCCTCACCAAACAGAGC	GTTAACTTGATCCAAAGCTCTGAG
PFKFB3	CAACTCCCCAACCGTGATTGT	TGAGGTAGCGAGTCAGCTTCT
GLUT1	CGGGCCAAGAGTGTGCTAAA	TGACGATACCGGAGCCAATG
LDHA	CAAAGACTACTGTGTAACTGCGA	TGGACTGTACTTGACAATGTTGG
β-actin	GTGACGTTGACATCCGTAAAGA	GCCGGACTCATCGTACTCC

## Data Availability

The original contributions presented in this study are included in the article material. Further inquiries can be directed to the corresponding author.

## Supplementary Materials

The following supporting information can be downloaded at https://www.mdpi.com/article/10.3390/nu17172800/s1, Figure S1: Ultrasound data are presented as mean ± standard deviation (SD) for each experimental group.
